# Cat exposure and asthma outcomes in a cohort of children with asthma and allergy

**DOI:** 10.3389/falgy.2026.1840756

**Published:** 2026-06-10

**Authors:** Resthie R. Putri, Cecilia Lundholm, Anna Hedman, Mwenya Mubanga, Hanna Karim, Jon R. Konradsen, Catarina Almqvist

**Affiliations:** 1 Department of Medical Epidemiology and Biostatistics, Karolinska Institutet, Stockholm, Sweden; 2 Strategic Information Unit, The Centre for Infectious Disease Research in Zambia, Lusaka, Zambia; 3 Pediatric Allergy and Pulmonology Unit, Astrid Lindgren Children’s Hospital, Karolinska University Hospital, Stockholm, Sweden

**Keywords:** allergic rhinitis, allergy, asthma, asthma exacerbation, cat, cat exposure, cat ownership, pediatric asthma

## Abstract

**Introduction:**

The impact of current pet exposure on children with allergic asthma is not yet fully understood. This study aimed to investigate the association between cat exposure and asthma outcomes in children with established asthma and allergy.

**Methods:**

A population-based cohort study using Swedish national health, sociodemographic, and quality registers was conducted. We included 30 277 children aged 4–17 years (born 2006–2020), with validated diagnoses of asthma and allergy, who had asthma care during the two years preceding exposure assessment. Cat exposure was defined as parental cat ownership recorded in the National Cat Register in 2023. Asthma outcomes, assessed during 2023–2024, included: asthma exacerbation and moderate-to-severe asthma. In a subset with available data (*n* = 1428), Forced Expiratory Volume in 1 Second (FEV_1_) and asthma control test (ACT) were evaluated. Odds ratios (ORs) were adjusted for sex, age, initial asthma severity, baseline airway allergy severity, parental asthma and allergy, population density, parental birth country, and socioeconomic status.

**Results:**

Of the study population (median age: 9.5 years, 39% females), 9.4% (*n* = 2,862) had cat exposure. Initial asthma severity was comparable between the cat-exposed and non-exposed groups (19.5% vs. 20.6% with moderate-to-severe asthma). Asthma exacerbation occurred in 3.3% of the cat-exposed group vs. 3.5% of the non-exposed group (adjusted OR: 1.12, 95% CI: 0.90–1.39). Moderate-to-severe asthma was observed in 9.6% vs. 10.1% (adjusted OR: 0.96, 0.84–1.10). No significant differences were found in ACT or FEV₁ z-scores. Within the cat-exposed group, no association between the number of cats, cat sex, or age and asthma outcomes was observed.

**Conclusion:**

In this cohort of children with asthma and allergy, no association between cat exposure and asthma exacerbations, severity, lung function, or asthma control was observed. These findings suggest that cat exposure may not adversely affect asthma outcomes in this population.

## 1 Introduction

Asthma remains one of the most common non-communicable diseases in childhood (1), and environmental factors are recognized as important aspects in asthma management (2). Among the environmental exposures, contact with companion animals receives considerable attention due to the common presence of furry animals in the home (3). Although furry animals are often assumed to have similar effects on asthma outcomes, different species produce distinct allergens and interact with humans in different ways (4). These differences can lead to varied impacts on asthma morbidity (5–8).

At least a quarter of European households own a cat (3). In clinical practice, families of children with asthma frequently encounter questions about managing cat exposure. Previous studies on cat exposure have focused primarily on the effect of early cat exposure on the development of asthma and allergic rhinitis (9–11). Previous studies on the impact of current cat exposure among children with established asthma have reported mixed findings (7, 8, 12, 13). Several clinical and epidemiological studies suggest that cat exposure does not consistently worsen asthma outcomes in children (7, 8, 12). In some cohorts, no independent association between cat ownership and exacerbation has been observed, whereas sensitization or concurrent viral infection has appeared to play a more important role (8, 12). Most existing studies are constrained by relatively small or selective study populations (7, 12, 13) and reliance on self-reported outcomes (7, 8), which may compromise generalizability and introduce bias. Additionally, different characteristics of cats may influence the concentration of cat allergen *Fel d 1* in the home (14, 15), yet whether cat characteristics translate into clinically meaningful differences in asthma morbidity remains unknown.

The study aimed to investigate the association between cat exposure and asthma outcomes in children with established asthma and airway allergy. Specifically, we examined asthma exacerbation, asthma severity, asthma control test (ACT) score, and forced expiratory volume in one second (FEV_1_). Among children with cat exposure, we explored whether cat characteristics (number of cats, cat sex, and age in the household) were associated with asthma outcomes.

## 2 Materials and methods

### 2.1 Study design, population, and variables

A cross-sectional cohort study was conducted. The study population included all children born in Sweden between 2006 and 2020, who were 4–17 years of age with established asthma and airway allergy. Asthma and allergy diagnoses were ascertained separately using validated algorithms for asthma (16) and allergic rhinitis (17, 18) based on diagnosis and medication codes (Supplementary Table S1). The inclusion criterion was documented asthma care (i.e., specialized care visit for asthma and/ or asthma medications) within two years preceding the exposure assessment in 2023. Children who had emigrated, died, or had missing parental sociodemographic data were excluded.

Cat exposure (yes/no) was defined as any recorded parental cat ownership at the baseline period (year 2023). Asthma outcomes were evaluated from the start of the baseline period through the end of the subsequent year, corresponding to a 24-month observation period. The main outcomes were:

1. Asthma exacerbation: defined as either an emergency or unplanned hospital/specialized care visit with asthma as the primary diagnosis, or ≥4 dispensations of short-acting beta 2-agonists (SABA) within 12 months.
2. Moderate-to-severe asthma: based on dispensed asthma medications, using adapted Global Initiative for Asthma (GINA) treatment steps (19). Moderate-to-severe asthma was defined as receiving more than one controller medication [e.g., a combination of inhaled corticosteroids (ICS) and leukotriene receptor antagonists (LTRA), or ICS/ LTRA and long-acting beta-2 agonists (LABA)], or if they were dispensed theophylline or biologics (details in Supplementary Table S2).

In a subset who had data of both asthma control test and spirometry during the outcome measurement period, additional outcomes were measured, namely:

1. Asthma control test (ACT) score, which was categorized as uncontrolled asthma if ACT ≤19.
2. Forced expiratory volume in 1 s (FEV_1_) standardized for age and height (z-score) based on the Global Lung Initiative (20).
3. The ratio between FEV_1_ and forced vital capacity (FEV_1_/FVC ratio) was additionally assessed among this subset with available FVC data.

Within the cat-exposed group, the association between the number of cats, cat sex, and cat age with the main asthma outcomes were assessed.

Other variables included as covariates:

- Child's sex: Male or female.
- Child's age: Measured on January 1st, 2023.
- Initial asthma severity: Categorized as mild or moderate-to-severe, based on dispensed asthma medications within one year after asthma onset.
- Severe allergic rhinitis at baseline: Defined using dispensed medications for allergic rhinitis that correspond to step 3 of the adapted European Forum for Research and Education in Allergy and Airway Diseases treatment algorithm (21) during the year before the exposure measurement (details in Supplementary Table S2).
- Parental asthma: Yes, if at least one parent had asthma.
- Parental allergy: Yes, if at least one parent had allergy.
- Population density: Calculated based on the number of inhabitants and the land area of the municipality where the child resided at baseline.
- Parents’ country of birth: Categorized as Nordic countries, other European countries (excluding Nordics), and non-European countries, assessed separately for each parent.
- Parental education: Defined as the highest attained education of either parent at baseline, categorized as compulsory school (≤9 years), high school (≈12 years), and university level (>12 years).

These variables were chosen *a priori* based on previous literature and a directed acyclic graph (Supplementary Figure S1). In addition, dog exposure (yes/no) was included for sensitivity analysis and was defined as any recorded parental dog ownership at baseline.

The Regional Ethical Review Board in Stockholm approved the study (Dnr 2018/1697-31/1; 2023-03916-02) and waived the requirement for informed consent.

### 2.2 Data sources

The Swedish national health and sociodemographic registers and a quality register were linked by Statistics Sweden and the National Board of Health and Welfare through the unique personal identity numbers. The Total Population Register and the Medical Birth Register were used to identify children born in Sweden (22, 23). Parental cat ownership was identified from the National Cat Register held by the Swedish Board of Agriculture. This cat register was established in 2023 (24), and registration is mandatory for all cats born in 2008 or later (25). Data on specialized-care diagnoses of asthma and allergy and on unplanned or emergency asthma visits were obtained from the National Patient Register (26). Diagnoses were identified using ICD-10 (International Classification of Diseases, 10th Revision) codes J45 and J46 for asthma, and J30 and J31.1 for allergic rhinitis, with full details provided in Supplementary Table S1. Unplanned or emergency visits were identified using the visit type recorded in the register. Data on prescriptions for asthma and allergy medications, across all levels of care, were obtained from the Prescribed Drug Register (27). The medications were identified using ATC codes (Anatomical Therapeutic Chemical Classification System), details specified in Supplementary Tables S1, S2. ACT, FEV_1_ and FVC data were obtained from the Swedish National Airway Register, which records patients with asthma at new registrations and follow-up visits from all levels of healthcare across Sweden (28). As most children had an established asthma diagnosis prior to the outcome measurement period, these data were likely obtained during follow-up visits. Parental countries of birth and population density based on the municipality of residence were taken from the Total Population Register. The parents’ highest attained education was obtained from the longitudinal integrated database for health insurance and labor market studies. Parental dog ownership was identified from the National Dog Register from the Swedish Board of Agriculture and the dog register from the Swedish Kennel Club. Further details about the data sources are in Supplementary Table S3.

### 2.3 Statistical analysis

Descriptive analyses were presented as counts and proportions for categorical variables and as medians, first quartiles (Q1), and third quartiles (Q3) for continuous variables. For each main outcome, a separate logistic regression was performed. Unadjusted odds ratios (OR), OR adjusted for all covariates, and their 95% confidence intervals were calculated. In a subset of the study population with available ACT and FEV_1_ data, the association of cat exposure with FEV_1_ and uncontrolled asthma was assessed using linear and logistic regression, respectively. In individuals from this subset with available FEV₁/FVC ratio data, the association between cat exposure and the FEV₁/FVC ratio was examined using linear regression. Within the cat-exposed group, the associations between cat characteristics and each main outcome were examined using logistic regression.

A sensitivity analysis was conducted to assess the association between cat exposure and asthma outcomes after excluding individuals with dog exposure. We also performed a sensitivity analysis to assess the impact of potential exposure misclassification (29) on the association between cat exposure and the main outcomes. As the National Cat Register was recently established (24), a substantial proportion of cat owners may not yet have registered their cats, leading to under-recording of cat ownership. We developed three different scenarios with varying distributions for the sensitivity of cat exposure classification. High specificity was assumed, given that false-positive exposure was expected to be uncommon. Parental registration as a cat owner is a strong indicator of true cat ownership, making misclassification of exposed children unlikely. For each scenario, a probabilistic bias analysis incorporating misclassification error was conducted on the summary level (30). This approach incorporates uncertainty in the assumed sensitivity and specificity, producing a distribution of bias-adjusted ORs. We generated this distribution using 1,000 repetitions and presented the median bias-adjusted OR, 2.5th and 97.5th percentiles. All statistical analyses were performed using Stata (version 19).

## 3 Results

### 3.1 Study population and characteristics

Of 32,010 children with asthma and allergy who remained having asthma within two years prior to exposure assessment, 1,733 were excluded due to migration (*n* = 1,115), death (*n* = 40), or missing parental data (*n* = 578). The final study population included 30,277 children [median age: 9.5 (6.2, 13.4) years, 38.6% females]. Among them, 2,862 children (9.4%) had cat exposure.

A higher proportion of females was observed in the cat-exposed group compared with the non-exposed group (43.2% vs. 38.1%). Initial asthma severity was similar between groups (moderate-to-severe asthma: 19.5% in the cat-exposed group vs. 20.6% in the non-exposed group). Severe allergic rhinitis at baseline was less common in the cat-exposed group 11.5% vs. 14.2% in the non-exposed group. Comparable proportions of parental asthma (43.9% vs. 44.9%) and parental allergy (77.1% vs. 77.3%) were observed, Table 1.

**Table 1:** Characteristics of the study population .

Characteristics	No cat	Cat	Total
	*N* = 27 415	*N* = 2 862	*N* = 30 277
Sex			
Males	19,961 (61.9)	1,625 (56.8)	18,586 (61.4)
Females	10,454 (38.1)	1,237 (43.2)	11,691 (38.6)
Age (years)	9.5 (6.1, 13.3)	10.5 (6.8, 13.9)	9.5 (6.2, 13.4)
Initial asthma severity^a^			
Mild	21,763 (79.4)	2,303 (80.5)	24,066 (79.5)
Moderate-to-severe	5,652 (20.6)	559 (19.5)	6,211 (20.5)
Severe allergic rhinitis at baseline^b^			
No	23,514 (85.8)	2,533 (88.5)	26,047 (86.0)
Yes	3,901 (14.2)	329 (11.5)	4,230 (14.0)
Parental asthma^c^			
No	15,108 (55.1)	1,606 (56.1)	16,714 (55.2)
Yes	12,307 (44.9)	1,256 (43.9)	13,563 (44.8)
Parental allergy^d^			
No	6,222 (22.7)	656 (22.9)	6,878 (22.7)
Yes	21,193 (77.3)	2,206 (77.1)	23,399 (77.3)
Population density (/km^2^)	455 (187, 1,216)	638 (269, 1,270)	468 (187, 1,221)
Father's country of birth			
Nordic	21,313 (77.7)	2,572 (89.9)	23,885 (78.9)
Europe except Nordic	1,762 (6.4)	113 (3.9)	1,875 (6.2)
Others	4,340 (15.8)	177 (6.2)	4,517 (14.9)
Mother's country of birth			
Nordic	21,627 (78.9)	2,638 (92.2)	24,265 (80.1)
Europe except Nordic	1,611 (5.9)	101 (3.5)	1,712 (5.6)
Others	4,177 (15.2)	123 (4.3)	4,300 (14.3)
Parental education			
Compulsory	707 (2.6)	44 (1.5)	751 (2.5)
High school	8,653 (31.6)	1,009 (35.3)	9,662 (31.9)
University level	18,055 (65.9)	1,809 (63.2)	19,864 (65.6)

Categorical variables are presented as *n* (%). Continuous variables are presented as median (Q1, Q3).

a Categorized based on adapted Global Initiative for Asthma (GINA) treatment steps (19).

b Defined using dispensed medications for allergic rhinitis that correspond to step 3 of the adapted European Forum for Research and Education in Allergy and Airway Diseases treatment algorithm (21).

c Identified according to a validated algorithm for asthma using diagnosis and medication codes (16).

d Identified according to a validated algorithm for allergic rhinitis using diagnosis and medication codes (17, 18).

### 3.2 The association between cat exposure and asthma outcomes

**Figure 1:**
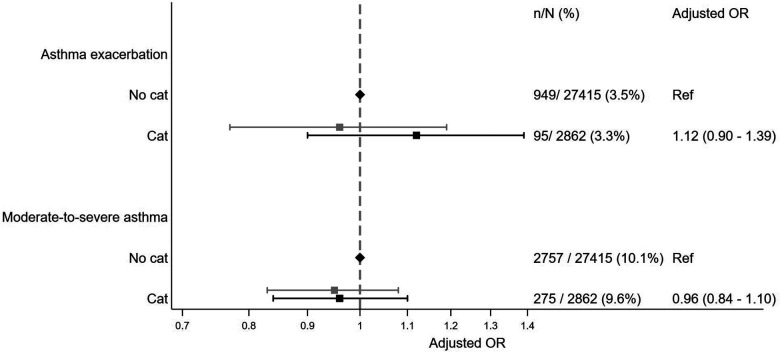
Association between cat exposure and asthma outcomes. Unadjusted odds ratios (ORs) and 95% confidence intervals (CIs) are shown in grey, and adjusted ORs and 95% CIs are shown in black. All ORs and CIs were estimated using logistic regression. ORs were adjusted for sex, age, initial asthma severity, allergic rhinitis severity at baseline, parental asthma, parental allergy, population density, parental education, and parental country of birth. CI, confidence intervals; OR, odds ratio.

Asthma exacerbation occurred in 3.3% of the cat-exposed group and 3.5% of the non-exposed group. Moderate-to-severe asthma was present in 9.6% of the cat-exposed group compared to 10.1% in the non-exposed group. No significant association was found between cat exposure and the asthma outcomes, Figure 1. No effect modification of the association by age or sex was seen (*p* for interaction = 0.42 and 0.44, respectively).

**Figure 2:**
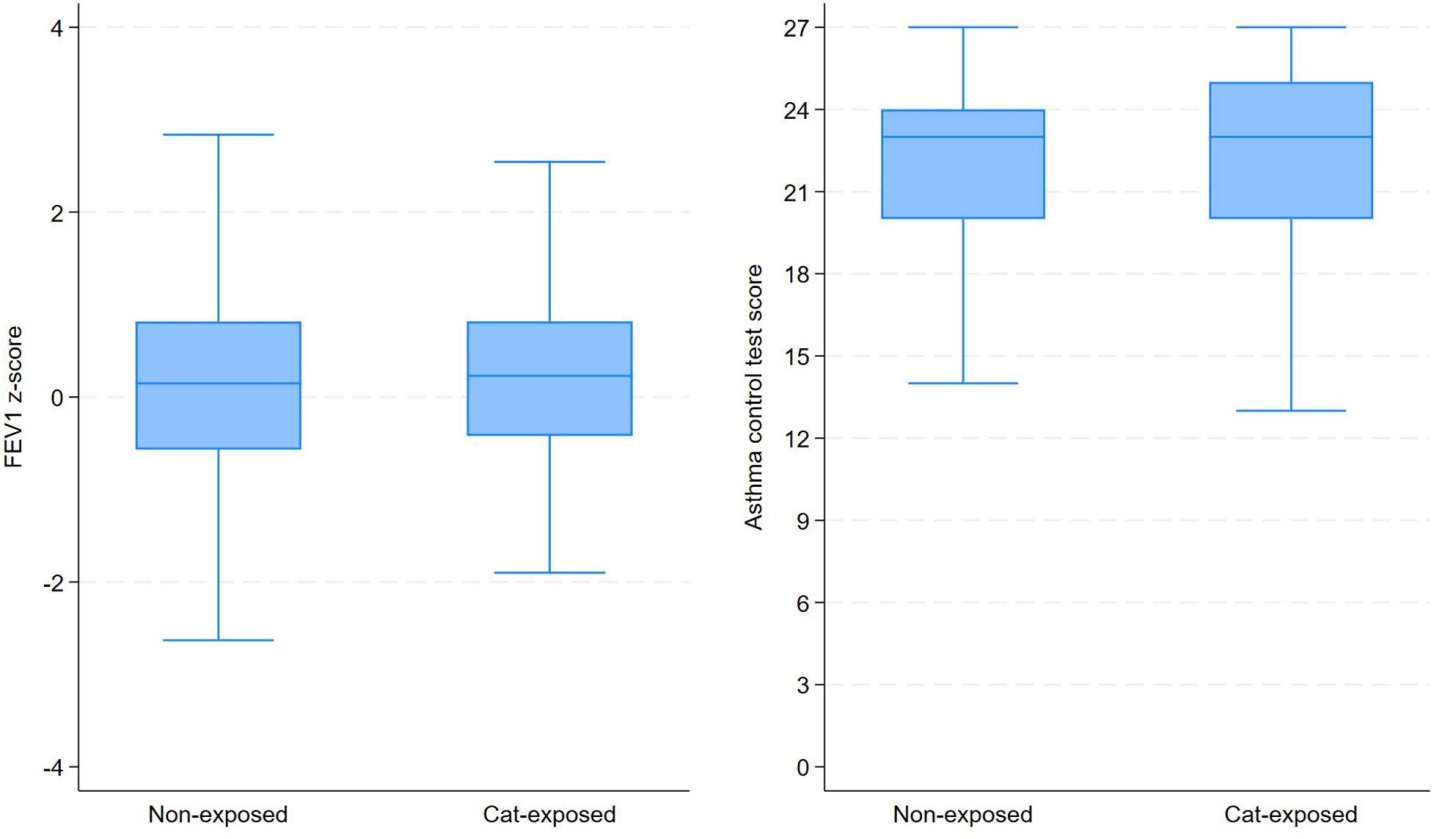
Boxplots of FEV_1_ z-score (left) and ACT score (right) by cat exposure group (cat-exposed, *n* = 97; non-exposed, *n* = 1,331). Horizontal lines inside the boxes represent the median FEV_1_ z-score or ACT in each group. Boxes extend from the 25th percentile to the 75th percentile of the values. The whiskers represent the 5th and 95th percentiles of the values. ACT, asthma control test; FEV1, forced expiratory volume in one second.

Of the study population, 1,428 children (97 of them with cat exposure) had both ACT score and FEV_1_ data during the outcome measurement period. Compared to the overall study population, this subset was older [median: 11.7 (9.1, 11.7) years], had a higher proportion of moderate-to-severe asthma at onset (27.2%), and a higher proportion of severe allergic rhinitis at baseline (28.8%). The distribution of sex, parental asthma, and parental allergy was comparable to that of the overall study population. In this subset, the proportion of moderate-to-severe asthma at onset was 23.7% (23/97) in the cat-exposed group and 27.5% (366/1331) in the non-exposed group. Characteristics of the subpopulation with ACT and FEV_1_ are shown in Supplementary Table S4. The proportion of uncontrolled asthma appeared slightly higher in the non-exposed group (22.3%) compared to the cat-exposed group (16.5%), yet no significant association was seen (adjusted OR: 0.70, 95% CI: 0.40–1.22). Figure 2 shows that the distributions of ACT and FEV-_1_ z-score were similar between the cat-exposed and non-exposed groups. Consistently, adjusted linear regression found no significant difference in mean FEV_1_ z-score between groups (*β* = −0.09, 95% CI: −0.37 to 0.20). Of this subset, 1,253 individuals had available FEV_1_/ FVC ratio data, and no significant difference in mean FEV_1_/ FVC ratio was observed between cat-exposed and non-exposed groups (*β* = −0.01, 95% CI: −0.02 to 0.005). The distribution of the FEV_1_/ FVC ratio is presented in Supplementary Figure S2.

#### 3.2.1 Cat characteristics and asthma outcomes

Within the cat-exposed group, asthma exacerbation and asthma severity did not differ by the number of cats, cat sex, or cat age (Table 2).

**Table 2:** Cat characteristics and asthma outcomes among children with cat exposure (*N* = 2,862).

Cat characteristics	Asthma exacerbation		Moderate–to–severe asthma	
	*n* (%)	Adjusted OR (95% CI)	*n* (%)	Adjusted OR (95% CI)
Number of cats				
1 cat	52 (3.2)	Ref	156 (9.6)	Ref
≥2 cats	43 (3.5)	0.97 (0.75–1.25)	119 (9.6)	1.07 (0.70–1.61)
Had a male cat				
No	33 (3.3)	Ref	92 (9.3)	Ref
Yes	62 (3.3)	1.03 (079–1.35)	183 (9.8)	0.97 (0.63–1.50)
Had a female cat				
No	38 (3.3)	Ref	110 (9.6)	Ref
Yes	57 (3.3)	1.00 (0.77–1.30)	165 (9.6)	1.01 (0.66–1.53)
Cat's age				
<2 years	40 (3.1)	Ref	117 (9.0)	Ref
≥2 years	55 (3.5)	1.14 (0.75–1.73)	158 (10.1)	1.17 (0.91–1.52)

CI, confidence intervals; OR, odds ratio.

ORs were estimated using logistic regression and adjusted for sex, age, initial asthma severity, allergic rhinitis severity at baseline, parental asthma, parental allergy, population density, parental education, parental country of birth.

#### 3.2.2 Sensitivity analyses

Similar null associations between cat exposure and asthma outcomes were observed after excluding those with dog exposure, Supplementary Table S5. The results for bias analysis for potential exposure misclassification are presented in Table 3. The 2.5th to 97.5th percentile for the bias-adjusted ORs included ORs both above and below 1 across all three scenarios, indicating uncertainty of the direction of an association.

**Table 3:** Results of probabilistic bias analysis of exposure misclassification under three sensitivity scenarios .

Sensitivity of cat exposure classification				Bias–adjusted OR, median (2.5th – 97.5th percentile)	
Min	m1	m2	Max	Asthma exacerbation	Moderate–to–severe asthma
0.2	0.3	0.3	0.4	0.89 (0.56–1.11)	0.88 (0.54–1.03)
0.4	0.5	0.5	0.6	0.90 (0.58–1.13)	0.88 (0.56–1.03)
0.5	0.6	0.6	0.7	0.90 (0.58–1.15)	0.89 (0.52–1.03)

min, minimum; m1, mode 1; m2, mode 2; max, maximum; OR, odds ratio.

Sensitivity and specificity of exposure classification were assumed to follow trapezoidal distributions. Misclassification was assumed to be non-differential. The specificity distribution was identical across all scenarios (min = 0.90, m1 = 0.95, m2 = 0.95, max = 1.00). Bias-adjusted ORs (median, 2.5th percentile, and 97.5th percentile) were estimated using probabilistic bias analysis accounting for both systematic and random error (30).

## 4 Discussion

Among children with asthma and allergy included in this study, no significant associations were observed between household cat exposure and either asthma exacerbation or moderate-to-severe asthma. In the subset with ACT and FEV₁ data, neither ACT scores nor FEV₁ differed significantly by cat exposure. Furthermore, within the cat-exposed group, no association was observed between the number of cats, cat sex, or cat age and asthma outcomes.

Several previous studies have examined the association between cat allergen exposure and pediatric asthma outcomes and demonstrated a null or modest association. A study of 300 children with asthma in the United States reported a modest association between elevated cat allergen *Fel d 1* levels and increased risk of severe asthma—as measured by parent-reported medication use over 12 months—among sensitized children (7). Notably, the study did not demonstrate a clear dose–response relationship; children with moderately elevated *Fel d 1* levels exhibited a higher prevalence of severe asthma than those with high levels (7). Similarly, a study of 84 children with asthma treated in a tertiary hospital in England found no independent effect of cat ownership on asthma exacerbations (12). Instead, an increased risk emerged only when cat ownership coincided with the presence of viral respiratory pathogens (12), suggesting that cat exposure alone may be insufficient to provoke exacerbations. Moreover, a large cross-sectional study of children and adults in the United States indicated that exacerbations were mainly associated with sensitization to cat, not with cat exposure itself (8). Prior studies have generally relied on single indicators, limiting the ability to capture the multidimensional nature of asthma outcomes. In contrast, our study incorporated multiple parameters of outcome (i.e., event of asthma exacerbation, asthma severity based on medications, ACT, FEV_1_, and FEV_1_/FVC ratio) and consistently found no obvious differences between children exposed and unexposed to cats. A study of 401 schoolchildren in Sweden provides additional context; following the summer holiday, children without regular cat contact who returned to classrooms with many cat-owning classmates had reductions in peak expiratory flow and increased asthma symptoms during a three-week observation (13). Taken together, the available evidence indicates that while sudden or high-level cat allergen exposure may trigger short-term symptom worsening, persistent exposure to cat allergens may not worsen asthma outcomes in children with established asthma and allergy.

The null association between cat exposure and asthma outcomes observed in the present study should not be extrapolated to other furry animals. A population-based study showed that dog exposure was associated with increased odds of asthma exacerbation among sensitized individuals, while no such association was observed for cat exposure (8). Similarly, we have shown in a national cohort of children with asthma and allergy that continuous dog exposure was associated with a slightly higher risk of asthma exacerbation and moderate-to-severe asthma (31), whereas in the current study, which focused on cat exposure, we found no evidence of an association. Hence, general advice on the avoidance of all furry animals may not be uniformly beneficial for children with allergic asthma.

Several mechanisms may help explain the null association between cat exposure and asthma outcomes observed in the present study. High exposure to cats, such as via cat ownership, appears to be associated with lower sensitization, partly because allergen exposure may promote the production of IgG antibodies that can block IgE-mediated allergic inflammation (32, 33). In addition, exposure to some animals (e.g., livestock, cat) may influence gut and airway microbiome in ways that reduce inflammation and airway hyperreactivity (34). Moreover, children without household cats are still exposed to cats to some extent, given that cat allergens are highly ubiquitous in community settings—schools, public transport, and even homes without cats often contain measurable cat allergens (35). Although initial asthma severity, parental asthma, and parental allergy were comparable between children with and without cat exposure, the cat-exposed group had a lower proportion of severe allergic rhinitis at baseline. Thus, residual confounding by indication (i.e., parents avoiding owning cats because of the child's allergy) cannot be fully ruled out.

Some evidence indicates that male cats produce higher levels of allergens (14) and that older cats tend to have lower allergen levels (15). Nevertheless, the present study did not observe any effect of number of cats, cat sex, or age on asthma exacerbation and severity. This indicates that any differences in allergen production related to these factors, if present, may not be sufficient to influence asthma outcomes.

The strength of the study includes the use of a national cohort of children with established asthma and allergy, and the ability to capture household cat ownership in a large population. This enhances the external validity of the findings. We also combined both objective measurements and self-reported measurements of the outcomes, allowing a comprehensive picture of how cat exposure may affect different dimensions of asthma outcomes. Nevertheless, several limitations should be acknowledged. Although all children in the study population had established allergies, data on allergen-specific IgE-mediated sensitization were unavailable. Given that degree and pattern of sensitization are associated with asthma severity (36), this lack of information may have resulted in residual confounding. In addition, the lack of information on patterns of mono-vs. polysensitization precluded more refined analyses, which may have important clinical relevance. Information on cat ownership prior to the study baseline and the duration of ownership was unavailable. In addition, among those individuals classified to have cat exposure, the indoor vs. outdoor status of the cat was not available. In Sweden, approximately 30% of household cats are outdoor cats (37). Nevertheless, outdoor cats in Sweden typically also spend considerable time indoors, even more so during the winter period, making the exposure likely irrespective of indoor–outdoor status. The National Cat Register used to identify exposure does not yet have full coverage, and some children with cat exposure may therefore have been misclassified as non-exposed. Although this issue was evaluated in the sensitivity analysis, the bias-adjusted estimates included both positive and negative ORs, leaving the underlying association uncertain. Variations in cat-keeping practice, cultural attitudes toward cats, weather, and climatic conditions across countries may limit the generalizability of our findings. Furthermore, as this study focused on allergic asthma, for which cat exposure is most biologically relevant, our findings may not be fully generalizable to non-allergic asthma phenotypes.

Among children with asthma and allergy, no significant association of cat exposure with asthma exacerbation, asthma severity, lung function, or asthma control was observed. This is the first large-scale epidemiological study to investigate the association in pediatrics population. Replication of these findings in different populations is warranted.

## Data Availability

The data analyzed in this study is subject to the following licenses/restrictions: The data supporting this study were obtained from third-party sources and cannot be made publicly available due to Swedish data protection and storage regulations. The original data are held by the Swedish National Board of Health and Welfare and Statistics Sweden. Access to these data requires approval from the Swedish Ethical Review Authority, followed by a formal application to the respective register holders. Researchers who obtain the necessary ethical approval may request access to the original register data directly from the register holders. Requests to access these datasets should be directed to The Swedish National Board of Health and Welfare, socialstyrelsen@socialstyrelsen.se; Statistics Sweden, scb@scb.se.
