# Supplementary material for: Cat exposure and asthma outcomes in a cohort of children with asthma and allergy
Source: Front Allergy. 2026 Jun 10;7:1840756. doi: 10.3389/falgy.2026.1840756 (PMC13290962; doi:10.3389/falgy.2026.1840756)
Supplement: Supplementary file 1 [file Datasheet1.docx]

Supplementary Material

Contents

[Table S1. Diagnosis and medication codes for asthma and allergic rhinitis 2](#_Toc227919650)

[Table S2. Medication codes for capturing severity of asthma and airway allergy 3](#_Toc227919651)

[Figure S1. Simplified directed acyclic graph of the investigated association in a population of children with asthma and allergy 4](#_Toc227919652)

[Table S3. Data sources 5](#_Toc227919653)

[Table S4. Characteristics of the subpopulation who had asthma control test and spirometry data 7](#_Toc227919654)

[Figure S2. Boxplot of FEV_1_/ FVC ratio by cat exposure groups 8](#_Toc227919655)

[Table S5. Association between cat exposure and asthma outcomes after excluding individuals with dog exposure 9](#_Toc227919656)

# Table S1. Diagnosis and medication codes for asthma and allergic rhinitis

| Asthma (1) | Allergic rhinitis (2) |
| --- | --- |
| **ICD-10 codes**  J45 Asthma  J46 Status asthmaticus  **ATC codes**  R03BA Glucocorticoids, inhalants  R03AC Selective beta-2-adrenoreceptor agonists, inhalants  R03AK Adrenergic in combination with corticosteroids or other drugs, excluding anticholinergics, inhalants  R03DC Leukotriene receptor antagonists  **Asthma diagnosis criteria**  Asthma diagnosis was ascertained using a validated algorithm (1). Briefly, for individuals aged 4.5 years or above, an individual has to have (a) at least a diagnosis of asthma based on ICD-10 codes, or (b) at least 2 dispensed of inhaled glucocorticoids , combination inhalants, or leukotriene receptor antagonists, or (c) at least 3 dispenses of inhaled beta-2-adrenoreceptor agonists, inhaled glucocorticoids, combination inhalants, or leukotriene receptor antagonists within a year. For individuals aged below 4.5 years; to ascertain asthma diagnosis, an individual has to fulfil criterion (a) above and either criterion (b) or criterion (c). | It was ascertained using the previous algorithm (2) by fulfilling 1 of the 4 criteria below.  **Criterion 1**. ≥1 ICD-10 codes:  J30 Allergic rhinitis and vasomotor rhinitis  J31.0 Chronic rhinitis  **Criterion 2.** ≥2 dispensed prescriptions of ATC codes:  R01AD01 – R01AD60 nasal preparation of corticosteroid  *And*  Without any of the ICD-10 codes below:  J33 Nasal polyps  J01 Acute sinusitis  J32 Chronic sinusitis  **Criterion 3**. ≥2 dispensed prescriptions of ATC codes:  R06A Antihistamines for systemic use  *And*  Without any of the ICD-10 codes below:  L29 Pruritus  DL50 Allergic urticaria  **Criterion 4**. ≥1 dispensed prescription of ATC code:  V01A Specific immune therapy, allergen subtract therapy  S01GX Medication for allergic conjunctivitis |

ICD-10 codes were obtained from the National Patient Register. ATC codes were obtained from the Prescribed Drug Register.

References:

1. Örtqvist, A.K., Lundholm, C., Wettermark, B., Ludvigsson, J.F., Ye, W. and Almqvist, C. (2013), Validation of asthma and eczema in population-based Swedish drug and patient registers. Pharmacoepidemiol Drug Saf, 22: 850-860. https://doi.org/10.1002/pds.3465
2. Henriksen L, Simonsen J, Haerskjold A, et al. Incidence rates of atopic dermatitis, asthma, and allergic rhinoconjunctivitis in Danish and Swedish children. J Allergy Clin Immunol. 2015;136(2):360-6.e2. doi:10.1016/j.jaci.2015.02.003

# Table S2. Medication codes for capturing severity of asthma and airway allergy

| Asthma severity (1) | Airway allergy severity (2) |
| --- | --- |
| Asthma severity was classified by adapting the five treatment steps of the Global Initiative for Asthma Report.  Moderate-to-severe asthma was considered if the study participants was prescribed medications aligned with GINA steps 3–5, namely:   - Combination therapy of ICS (ATC: R03BA, R03AK) and LTRA (ATC: R03DC03) - ICS/LTRA in combination with LABA (ATC: R03AC12, R03AC13) - ICS/LTRA in combination with LAMA (ATC: R03BB04, R03AL06) - Biologic asthma medications (e.g., omalizumab; ATC: R03DX05) - Theophylline (ATC: R03DA04). | Airway allergy severity was classified by adapting a stepwise treatment approach. Severe airway allergy was considered if the study participant was prescribed allergic rhinitis medications aligned with treatment step 3, with steps 1–2 representing milder disease.  The treatment steps were defined as follows:  Treatment step 1  Prescribed either of the following:  Nasal corticosteroids (ATC: R01AD05, R01AD08, R01AD09, R01AD11, R01AD12), or  Non-sedating oral antihistamines (ATC: R06AX)  Treatment step 2  Prescribed either:  Fixed combination of nasal corticosteroids plus nasal antihistamines (ATC: R01AD58, R01AD59), or  Any two of the following three medication classes:  Nasal corticosteroids (ATC codes as above)  Non-sedating oral antihistamines (ATC: R06AX)  Ocular antihistamines (ATC: S01GX)  Treatment step 3 (Severe allergic rhinitis)  Severe allergic rhinitis was considered if the study participant was prescribed medications aligned with treatment step 3, namely:   - LTRA (ATC: R03DC03) - Ocular antihistamines (ATC: S01GX) - Short-course oral corticosteroids (ATC: H02AB01, H02AB02, H02AB04)   These step‑3 treatments were assessed in addition to the medications already included in steps 1–2. |

Abbreviations: ATC, Anatomical Therapeutic Chemical Classification System; ICS, Inhaled Corticosteroids; LABA, Long‑Acting Beta‑Agonist; LAMA, Long‑Acting Muscarinic Antagonist; LTRA, Leukotriene Receptor Antagonist.

References:

1. Global Initiative for Asthma. Global Strategy for Asthma Management and Prevention [Internet]. 2024. Available from: <https://ginasthma.org/2024-report/>
2. Hellings PW, Scadding G, Bachert C, Bjermer L, Canonica GW, Cardell LO, et al. EUFOREA treatment algorithm for allergic rhinitis. Rhinology journal. 2020 Dec;58(6):618–22.

# Figure S1. Simplified directed acyclic graph of the investigated association in a population of children with asthma and allergy


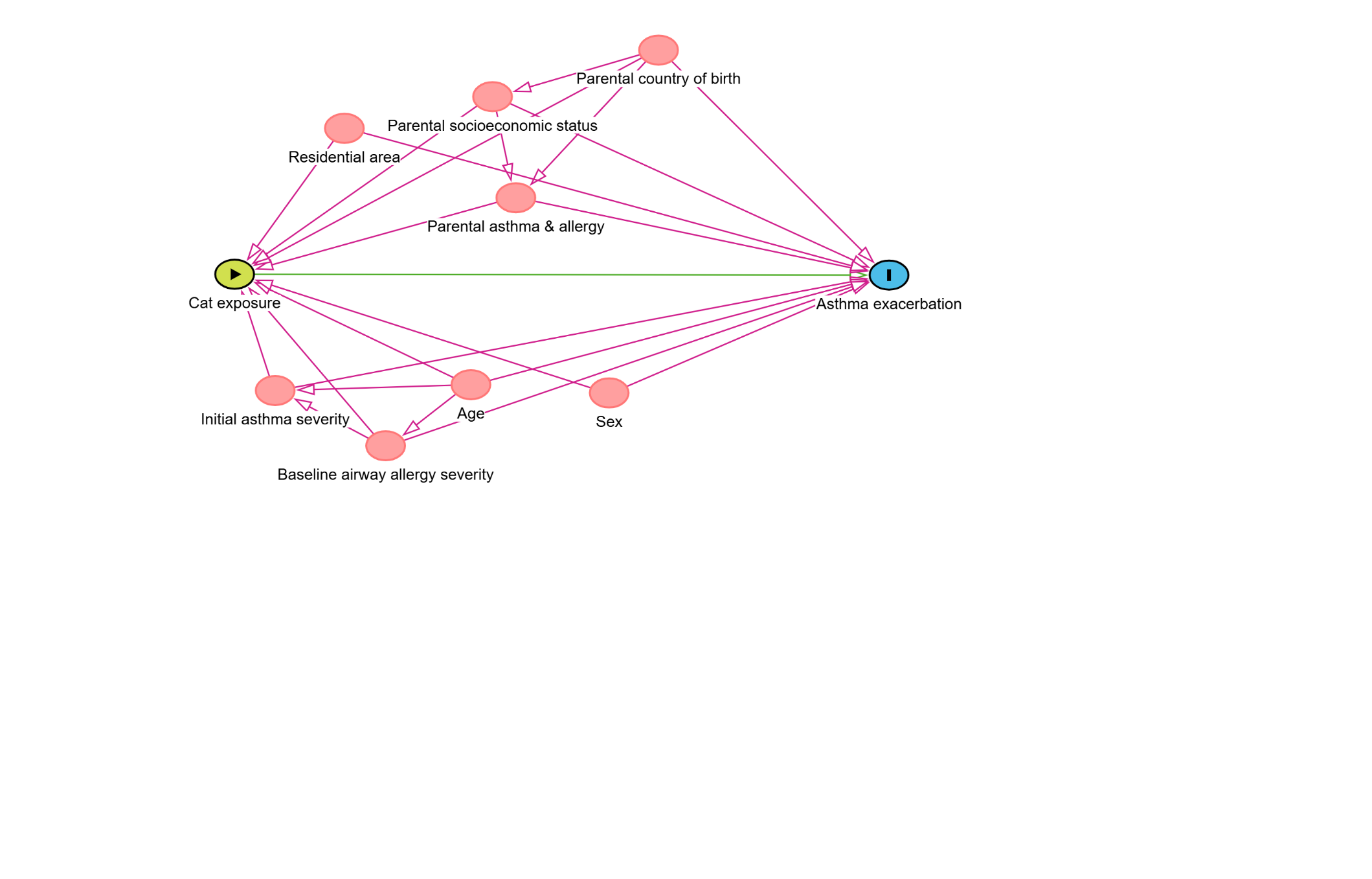


# Table S3. Data sources

| **Data source** | **Description** |
| --- | --- |
| Total Population Register (1) | Used together with the Medical Birth Register to identify all children born in Sweden and their biological parents, as well as to obtain data on residential area, any migration, and parents’ country of birth. The register began in 1968 and includes all individuals living in Sweden. |
| Medical Birth Register (2) | Established in 1973, this register contains information on nearly all births in Sweden (97–99% coverage). It includes maternal characteristics, pregnancy details, and perinatal outcomes. |
| National Cat Register (3) | Introduced in January 2023 and maintained by the Swedish Board of Agriculture. Based on the regulation, all cats born 2008 onward should be identified (via microchip or tattoo) and registered along with the owner’s information. |
| National Patient Register (4) | Captures diagnoses and procedures from inpatient care (nationwide since 1987) and outpatient specialist visits (since 2001). Reporting by specialised healthcare is mandatory, and most diagnoses recorded in the register have a high positive predictive value. |
| Prescribed Drug Register (5) | Started in 2005, this register contains records of all prescribed medications dispensed at pharmacies in Sweden. |
| Cause of Death Register (6) | Provides information on deaths and dates of death for all individuals living in Sweden. This register has been maintained since 1961 and is considered highly complete |
| Longitudinal Integrated Database for Health Insurance and Labor Market Studies (7) | Contains annual data on education, income, and employment for Swedish residents aged 16 years or older. This database was established in 1990. |
| Swedish National Airway Register (8) | Initiated in 2013, the register includes data of paediatric and adult patients with asthma from primary, secondary, and tertiary care across Sweden. Data of diagnostic or monitoring assessments (e.g., spirometry, blood test, symptom score) are recorded. |
| National Dog Register (9) | Introduced in 2001 and held by the Swedish Board of Agriculture. Since year 2001, it has been compulsory by law that all dog owners register their dogs. Variables available in the register include data of the dog owner, the dog identification chip or tattoo, if the dog has deceased and the date. |
| Dog register from the Swedish Kennel Club (10) | The digital database for dogs started in 1975. It records mainly purebred dogs and dogs actively involved in competition. The dog registration is voluntary. |

References:

1. Ludvigsson JF, Almqvist C, Bonamy AK, et al. Registers of the Swedish total population and their use in medical research. Eur J Epidemiol. 2016;31(2):125-136. doi:10.1007/s10654-016-0117-y
2. Cnattingius S, Källén K, Sandström A, et al. The Swedish medical birth register during five decades: documentation of the content and quality of the register. Eur J Epidemiol. 2023;38(1):109-120. doi:10.1007/s10654-022-00947-5
3. The Swedish Board of Agriculture. Identification and registration of cats. https://jordbruksverket.se/languages/english/swedish-board-of-agriculture/animals/identification-and-registration-of-animals/identification-and-registration-of-cats.
4. Everhov ÅH, Frisell T, Osooli M, et al. Diagnostic accuracy in the Swedish national patient register: a review including diagnoses in the outpatient register. Eur J Epidemiol. Published online March 27, 2025. doi:10.1007/s10654-025-01221-0
5. Wettermark B, Hammar N, Fored CM, et al. The new Swedish Prescribed Drug Register--opportunities for pharmacoepidemiological research and experience from the first six months. Pharmacoepidemiol Drug Saf. 2007;16(7):726-735. doi:10.1002/pds.1294
6. Brooke HL, Talbäck M, Hörnblad J, et al. The Swedish cause of death register. Eur J Epidemiol. 2017;32(9):765-773. doi:10.1007/s10654-017-0316-1
7. Ludvigsson JF, Svedberg P, Olén O, Bruze G, Neovius M. The longitudinal integrated database for health insurance and labour market studies (LISA) and its use in medical research. Eur J Epidemiol. 2019;34(4):423-437. doi:10.1007/s10654-019-00511-8
8. Stridsman C, Konradsen JR, Vanfleteren L, et al. The Swedish National Airway Register (SNAR): development, design and utility to date. Eur Clin Respir J. 2020;7(1):1833412. doi:10.1080/20018525.2020.1833412
9. The Swedish board of Agriculture. Identification and registration of dogs. https://jordbruksverket.se/languages/english/swedish-board-of-agriculture/animals/identification-and-registration-of-animals/identification-and-registration-of-dogs.
10. The Swedish Kennel Club. Olika hundregister - Registrerad i SKK? https://www.skk.se/aga-hund/uppgifter-om-din-hund-hos-skk/registrering/.

# Table S4. Characteristics of the subpopulation who had asthma control test and spirometry data

|  | No cat | Cat | Total |
| --- | --- | --- | --- |
|  | N = 1331 | N = 97 | N = 1428 |
|  |  |  |  |
| Sex |  |  |  |
| Males | 808 (60.7) | 55 (56.7) | 863 (60.4) |
| Females | 523 (39.3) | 42 (43.3) | 565 (39.6) |
| Age (years) | 8.9 (7.12, 11.5) | 13.3 (10.3, 15.0) | 11.7 (9.1, 14.4) |
| Initial asthma severity |  |  |  |
| Mild | 965 (72.5) | 74 (76.3) | 1039 (72.8) |
| Moderate-to-severe | 366 (27.5) | 23 (23.7) | 389 (27.2) |
| Severe allergic rhinitis at baseline |  |  |  |
| No | 948 (71.2) | 68 (70.1) | 1016 (71.2) |
| Yes | 383 (28.8) | 29 (29.9) | 412 (28.8) |
| Parental asthma |  |  |  |
| No | 688 (51.7) | 56 (57.7) | 744 (52.1) |
| Yes | 643 (48.3) | 41 (42.3) | 684 (47.9) |
| Parental allergy |  |  |  |
| No | 292 (21.9) | 21 (21.6) | 313 (21.9) |
| Yes | 1039 (78.1) | 76 (78.4) | 1115 (78.1) |
| Population density (/km^2^) | 187 (131, 447) | 344 (187, 1168) | 187 (130, 455) |
| Father's country of birth |  |  |  |
| Nordic | 984 (75.1) | 81 (84.4) | 1065 (75.7) |
| Europe except Nordic | 96 (7.3) | 5 (5.2) | 101 (7.2) |
| Others | 230 (17.6) | 10 (10.4) | 240 (17.1) |
| Mother's country of birth |  |  |  |
| Nordic | 1009 (75.9) | 81 (83.5) | 1090 (76.4) |
| Europe except Nordic | 86 (6.5) | 7 (7.2) | 93 (6.5) |
| Others | 235 (17.7) | 9 (9.3) | 244 (17.1) |
| Parental education |  |  |  |
| Compulsory | 25 (1.9) | 0 (0) | 25 (1.8) |
| High school | 322 (24.2) | 27 (27.8) | 349 (24.4) |
| University level | 984 (73.9) | 70 (72.2) | 1054 (73.8) |
|  |  |  |  |

# Figure S2. Boxplot of FEV_1_/ FVC ratio by cat exposure groups

Horizontal lines inside the boxes represent the median FEV_1_ / FVC ratio. Boxes extend from the 25^th^ percentile to the 75^th^ percentile of the values. The whiskers represent the 5^th^ and 95^th^ percentiles of the values.

# Table S5. Association between cat exposure and asthma outcomes after excluding individuals with dog exposure

|  | Asthma exacerbation | | | Moderate-to-severe asthma | | |
| --- | --- | --- | --- | --- | --- | --- |
|  |  | OR (95% CI) | |  | OR (95% CI) | |
|  | n/N | Unadjusted | Adjusted | n/N | Unadjusted | Adjusted |
| No cat | 777/ 22 930  (3.4%) | Ref | Ref | 2281/ 22 930  (9.9%) | Ref | Ref |
| Cat | 79/ 2322  (3.4%) | 1.00  (0.79 - 1.27) | 1.21  (0.95 - 1.54) | 225/ 2322  (9.7%) | 0.97  (0.84 - 1.12) | 0.98  (0.84 - 1.13) |
|  |  |  |  |  |  |  |

ORs and 95% CI were estimated using logistic regression and adjusted for sex, age, initial asthma severity, allergic rhinitis severity at baseline, parental asthma, parental allergy, population density, parental education, and parental country of birth.

Excluded from the analyses were individuals with parental dog ownership. Of the whole study population, the prevalence of parental dog ownership: 18.8% among cat-exposed group, and 16.4% among non-cat-exposed group.
